# Ever-never heard of ‘Malaysian Healthy Plate’: How many people know and practice this healthy eating concept?

**DOI:** 10.1371/journal.pone.0288325

**Published:** 2023-07-17

**Authors:** Mohamad Hasnan A., Norsyamlina C. A. R., Cheong S. M., Ruhaya S., Munawara P., Wan Shakira R. H., Shubash S. G., Khairul Hasnan A., Noor Ani A.

**Affiliations:** 1 Institute for Public Health, National Institutes of Health, Ministry of Health Malaysia, Shah Alam, Malaysia; 2 Universiti Malaysia Kelantan, Kota Bharu, Kelantan, Malaysia; University of Petra (UOP), JORDAN

## Abstract

**Introduction:**

The current and simpler healthy eating concept ‘Malaysian Healthy Plate’, was introduced in 2016. This study aims to determine the prevalence of ever-never heard, understanding, and practice of this concept and also the associated factors to never-heard of Malaysian Health Plate after three years of the implementation.

**Methodology:**

This study utilized the data from the National Health and Morbidity Survey (NHMS) 2019, a cross-sectional survey that applied two-stage stratified cluster sampling. Questionnaires pertained to ever heard, knowledge, and practised of Malaysian Healthy Plate were asked along with another questionnaire. Complex sample analysis was applied to fit with the study design.

**Results:**

Estimated 16.9 million (79.6%) adults in Malaysia were never heard of the Malaysian Healthy Plate Concept in 2019. Never heard of this concept was significantly associated with sex (men, aOR 0.265), ethnicity (other ethnicities, aOR 1.79), an education level (no education, aOR 9.59; primary, aOR 3.50; secondary, aOR 1.70), occupation (private, aOR 2.16; self-employed, aOR 2.31; retirees, aOR 1.69; student, aOR 2.31; unpaid workers, aOA 2.35; not working, aOR 2.86), non-diabetes aOR 1.43, and low fruit and vegetable intake person aOR 1.86. Meanwhile, another 20.4% or an estimated 4.3 million adults who were ever heard of Malaysian Healthy Plate. Among those who are ever heard, 80.8% (3.5 million) correctly interpreted or understood the concept and among those who ever heard and understood the concept, only 70.5% (2.5 million) have been practising it daily or occasionally.

**Conclusions:**

Four-fifth of adults never heard of the ‘Malaysian Healthy Plate’ and among those who ever heard, the understanding and practising of the concept were probably unsatisfactory. Multisectoral collaboration is an urge to fasten the promotion to spark awareness and educate the public to practice the concept to promote healthy eating and a healthier nation.

## Introduction

Finding An effective strategy for promoting healthy eating is always a big challenge to healthcare providers. Healthy eating messages were delivered to the public a utilising simple symbol, an interactive graphic, and a catchy tagline, or sometimes involving a public figure as a role model. Other countries such as the United States had introduced a new concept for healthy eating named MyPlate for replacing MyPyramid in 2011 [1]. According to a study conducted by the USDA’s Center for Nutrition Policy and Promotion, while most consumers had understood the key principles provided by MyPyramid, there was still a knowledge gap in calories and portions domains. According to their focus groups, consumers preferred a plate-shaped emblem since it sent a good message about healthy eating habits [2, 3]. The consumers too indicated they found the terms “Protein” and “Dairy” were more comprehensive than “Meat and Beans” and “Milk and Milk Products,” respectively [4].

After the launching of ’The Malaysian Food Pyramid Concept’ in the Malaysian Dietary Guideline in 2010, a simpler and latest concept which is the ‘Malaysian Healthy Plate Concept’ was introduced in late of the year 2016. This concept using the tagline ‘*SukuSukuSeparuh*’ or ’Quarter-Quarter-Half’ is a simple and practical guide in assisting the public to make healthy food choices [5]. Based on this concept, a plate is divided into two quarters and one half, and this helps to determine how much protein, grains, fruits, and vegetables should be consumed with each meal. The recommended portions for a healthy meal are a quarter for grains like rice or other cereals as well as to fill with whole grains, another quarter for proteins like chicken, meat, seafood, and bean products, and a half for fruits and vegetables [5, 6]. The concept is different from the Malaysian Food Pyramid, where there are five food groups inside with four-level in the pyramid and the five food groups come with the specific recommendation for servings [6].

The end goal of introducing this concept is to prevent the occurrence of non-communicable diseases (NCDs) such as hypertension, hypercholesterolemia, diabetes, anaemia, obesity, and certain types of cancer [7, 8]. It also the response from the Non-communicable Disease Division and Nutrition Division, Ministry of Health Malaysia to the results of the National Health and Morbidity Survey (NHMS) that reported the prevalence of non-communicable disease had increased among adults in Malaysia and healthy eating practices was also a concern. It was only 7.5% of adult Malaysians consumed sufficient fruits and vegetables in 2011 and the prevalence surprisingly reduced to 6.0% in 2015 [9, 10].

Starting the lunching of this Malaysia Health Plate in national level in 2016, the promotion expanded to lunching in state level, roadshow during nutrition month every April, promotion in mass media, talk by nutritionist/dietitian, distribution of education material to public and include this concept in programme such as ‘Healthy Cafetria’ and ‘Healthy Meal During Meeting. After 3 years this concept was introduced with many engagements and promotions on this Malaysia Health Plate Concept, and the coverage in population needs to be identified. Therefore, as the NHMS on the non-communicable disease was carried out in 2019, we included the objective to determine the prevalence of ever-never of the Malaysian Healthy Plate Concept among adults in Malaysia and its associated factors. All together, we also aim to determine the prevalence of knowledge and practice on this Malaysian Healthy Plate Concept among those who were aware of this healthy eating approach.

## Materials and methods

### Study design and sampling

This study is a part of the National Health and Morbidity Survey (NHMS) 2019 which is a nationwide community-based cross-sectional study. NHMS was conducted routinely annually and in 2019 it focused on Non-Communicable Diseases (NCDs) and Health Care Demand (HDC). The sample size was determined using the single proportion calculation, with adjustments to reflect the complex design, number of strata, and expected non-response rate to ensure the sample is enough to represent the adult population in Malaysia. Based on the proportion of 50% as this is the first-time assessment on the Malaysian Healthy Plate Concept is tested, type 1 error or alpha (α) is equal to 0.05, power (β) equal to 80%, design effect equal to 1.5, and the number of strata equal to 16, a total of 9,216 individual above 18 years old were required for analysis.

The study applied a multistage stratified random sampling design to ensure the selection of a nationally representative sample. Population data from the Department of Statistics Malaysia (DOSM) was used as the sampling frame. Sample selection starts at Enumeration Block (EB) to the Living Quarters (LQ) and finally to the individual residing in the living quarters. Details on the methodology of this survey were explained in the official technical report [11].

### Inclusion and exclusion criteria

This study included all participants aged 18 and above who remained in the selected living quarters for at least one month. Meanwhile, individuals under the age of 18 or adults who did not stay in the selected living quarters for at least one month or who were extremely severely ill at the time of data collection were excluded from this study. Ethics approval for the study was obtained from the Medical Research Ethics Committee (MREC), NCDs–Non-Communicable Diseases: Risk Factors and other Health Problems NMRR-18-3085-44207. All respondents signed the consent forms prior to taking measurements and participating in the interview sessions.

### Questionnaire and tool

Questionnaires pertained to ever-never heard, knowledge, and practice on the Malaysian Healthy Plate concept. To assess the ever-never heard, the respondents were asked ‘Have you ever heard about the Malaysian Healthy Plate or Quarter Quarter Half?’ with the binary responses ’Yes’ or ’No’. For knowledge or interpretation, the respondent was requested to interpret the type and portion of foods that should be filled on the plate. All answers provided by the respondents were required to be 100% accurate to indicate the respondents have adequate knowledge and understanding of the Malaysian Healthy Plate concept which is a quarter of the plate is rice or carbohydrate source, a quarter plate is fish or protein source and half of the plate vegetables and fruits. For the practice, the respondents were asked to respond how frequently he or she practices the concept either ’every day, ’sometimes’ or ’never’.

Other information also collected which 1) Sociodemographic characteristics; strata, sex, age, ethnicity, marital status, education level, occupation, and household income group based on the current classification from Household Income Survey, Department of Statistic Malaysia [12] (2) dietary practices; fruit and vegetable intake, (3) anthropometric measurement; weight, height, and waist circumference and (4) clinical data; anaemia, hypertension, diabetes, and hyper cholesterol.

The questionnaire and data from measurement were adapted in the Institute of Public Health survey application (survey creating system–SCS) without changing the structure, answer options and units of measurement. The application was installed on tablets and was used by trained research assistants during face-to-face interviews. All questionnaires, manual and system, were prepared in Malay and English and pretested before being used during data collection.

### Statistical analysis

Data were analysed using the IBM Statistical Package for the Social Sciences (SPSS) software for Windows, Version 26.0 by complex samples analysis module to ensure population representativeness. This complex sample analysis module determined the frequencies, prevalence of unknowing, proportion of knowledge, and proportion of practising Malaysian Healthy Plate. The Rao-Scott adjusted chi-square statistic was utilized for the test if the unknowing status and sociodemographic factors were independent. The relationships between unknowing and other characteristics were examined using complex sample logistic regression with 95% confidence intervals, a p-value of 0.05 significance level, and control for all possible confounding factors.

## Results

A total of 10,464 respondents were successfully recruited which represents about 21.3 million adults 18 years old in Malaysia as we applied the complex sample analysis. Complex sample frequencies analysis found the prevalence of never heard of the ‘Malaysian Healthy Plate’ was 79.6% or equal to 16.9 million adults in Malaysia. The comparison through Rao-Scott adjusted chi-square statistic found that never heard of Malaysian Healthy Plate was significantly higher among adults residing in rural, males, those aged above 60 years old, Chinese and other ethnicities, widow or divorced persons, those with lower education level, non-government staff and those earned lower household income as shown in Table 1.

**Table 1:** Prevalence of never heard of the ‘Malaysian Healthy Plate’ concept by sociodemographic characteristics of the respondents (N = 10464).

Socio-demographic characteristics	Never heard of Malaysian Healthy Plate				
	Unweighted Count	Estimated population	Prevalence (%)	95% CI	
				Lower	Upper
**Malaysia**	8116	16918567	79.6	78.0	81.1
**Strata**					
Urban	4785	13034952	78.5	76.6	80.4
Rural	3331	3883615	83.2*	81.0	85.3
**Sex**					
Male	4071	9475736	86.7*	85.0	88.2
Female	4045	7442831	72.0	69.8	74.1
**Age group**					
18–39 years old	3135	9164857	77.8	75.5	79.9
40–59 years old	2823	4990129	78.4	76.2	80.4
60+ years old	2158	2763581	88.7*	86.5	90.5
**Ethnicity**					
Malays	5001	8117524	74.6	72.7	76.4
Chinese	1132	3788851	83.1*	79.3	86.2
Indians	523	972156	78.4	73.5	82.6
Others	1460	4040036	88.3*	85.4	90.6
**Marital status**					
Single	1720	4929292	81.7	79.2	84.0
Married	5431	10693923	77.9	76.0	79.7
Widow/divorce	965	1295352	85.8*	82.7	88.4
**Edu. level**					
No formal	637	1097223	96.0*	93.7	97.5
Primary	2119	3712304	90.5*	88.3	92.3
Secondary	3885	8307993	80.3*	78.3	82.2
Tertiary	1454	3734663	66.6	63.5	69.6
**Occupation**					
Gov./semi gov.	520	896641	57.3	52.6	61.9
Private	2403	6688091	81.5*	79.1	83.6
Self-employed	1559	3083219	83.4*	80.4	86.1
Retirees	406	639538	80.6*	75.2	85.0
Student	201	570890	77.5*	70.6	83.3
Unpaid workers	1528	2665063	74.8*	71.0	78.2
Not working	1493	2366752	87.9*	85.1	90.3
**Threshold income**					
Bottom 40%	5357	10625271	81.7*	80.0	83.3
Middle 40%	1690	3952186	76.7	73.5	79.6
Top 20%	521	1307660	71.6	65.8	76.8

Edu.:education; Gov.: government

*p<0.05 for Rao-Scott adjusted chi-square statistic

Further analysis by complex sample logistic regression found that never heard of this concept was significantly associated with sex, where men were 2.67 times more compared to women. Other ethnicities were also found 1.79 times more likely to never hear of this concept compared to Malay ethnicity. For education level, those who had no formal education, primary school, and secondary school were 9.59, 3.50 and 1.70 times respectively to be more likely never heard of the ‘Malaysian Healthy Plate’ concept compared to those who had tertiary education level. Those who were employed in the non-government sector or unemployed were about 2 times more likely to never hear of this concept compared to government staff. Non-diabetic adults and those with low fruit and vegetable intakes were 1.43 and 1.86 times more likely to never hear of this concept as shown in Table 2.

**Table 2:** Factors associated with never heard of the ‘Malaysian Healthy Plate’ concept.

Variables	Never heard (%)	Logistic regression analysis	
		OR (95%CI)	aOR (95%CI)
**Location**			
Urban	78.5	1	1
Rural	83.2	1.36* (1.13, 1.64)	1.04 (0.84, 1.28)
**Sex**			
Male	86.7	2.53* (2.17, 2.93)	2.65* (2.18, 3.23)
Female	72.0	1	1
**Age group**			
18–39 years old	77.8	1	
40–59 years old	78.4	1.04 (0.88, 1.22)	1.08 (0.86, 1.37)
60+ years old	88.7	2.23* (1.80, 2.77)	1.39 (0.95, 2.03)
**Ethnicity**			
Malays	74.6	1	1
Chinese	83.1	1.67* (1.29, 2.17)	1.38 (0.98, 1.94)
Indians	78.4	1.24 (0.94, 1.62)	1.17 (0.85, 1.62)
Others	88.3	2.56* (1.97, 3.32)	1.79* (1.35, 2.37)
**Marital status**			
Single	81.7	1.27* (1.07, 1.51)	1.12 (0.88, 1.43)
Married	77.9	1	1
Widow/divorce	85.8	1.71* (1.35, 2.18)	1.06 (0.77, 1.46)
**Edu. Level**			
No formal	96.0	12.13*(7.25, 20.28)	9.59*(5.44, 16.93)
Primary	90.5	4.79* (3.71, 6.16)	3.50* (2.52, 4.85)
Secondary	80.3	2.05* (1.73, 2.42)	1.70* (1.40, 2.05)
Tertiary	66.6	1	1
**Occupation**			
Gov./semi-gov.	57.3	1	1
Private	82.5	3.28* (2.62, 4.12)	2.16* (1.62, 2.87)
Self-employed	87.4	3.75* (2.85, 4.92)	2.31* (1.66, 3.20)
Retirees	74.8	2.21* (1.69, 2.91)	1.69* (1.09, 2.62)
Student	80.6	3.09* (2.17, 4.39)	2.31* (1.27, 4.22)
Unpaid workers	77.5	2.57* (1.70, 3.89)	2.35* (1.66, 3.32)
Not working	87.9	5.44* (4.01, 7.37)	2.86* (1.86, 4.40)
**Threshold income**			
Bottom 40%	81.7	1.77* (1.34, 2.35)	1.06 (0.76, 1.48)
Middle 40%	76.7	1.30 (0.96, 1.78)	1.09 (0.78, 1.53)
Top 20%	71.6	1	1
**BMI Status**			
Normal	86.2	2.12* (1.44, 3.12)	1.56 (0.93, 2.61)
Underweight	81.7	1.51* (1.25, 1.83)	1.12 (0.85, 1.48)
Overweight	77.5	1.15 (0.96, 1.39)	1.01 (0.81, 1.26)
Obese	74.7	1	1
**Abdominal obesity**			
Yes	76.3	1	1
No	82.5	1.47* (1.26, 1.71)	1.06 (0.84, 1.34)
**Anemia status**			
Yes	75.1	1	1
No	80.2	1.35* (1.14, 1.58)	1.13 (0.95, 1.36)
**Hypertension status**			
Yes	81.6	1	1
No	78.7	0.83* (0.71, 0.99)	0.99 (0.81, 1.22)
**Diabetes**			
Yes	77.8	1	1
No	79.7	1.12 (0.93, 1.35)	1.43* (1.10, 1.87)
**Hyper-cholesterol**			
Yes	76.9	1	1
No	81.2	1.29* (1.12, 1.49)	1.11 (0.93, 1.33)
**Fruits & Vege intake**			
Adequate	69.3	1	1
Not-adequate	79.2	1.69* (1.12, 2.56)	1.86* (1.23, 282)

BMI: Body Mass Index; OR: Odd Ratio; aOR: adjusted Odd Ratio;

Gov.: government; Vege: vegetables

*Significant p<0.05 for complex sample logistic regression

**Fig 1:**
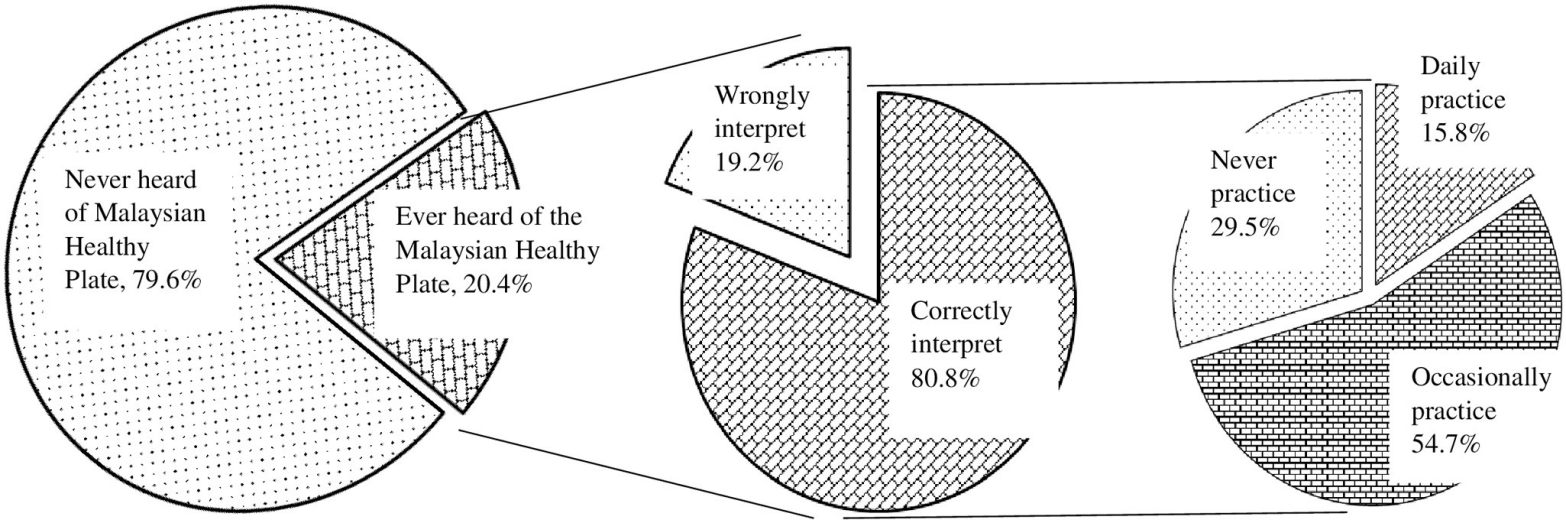
Status of ever heard, knowledge in interpretation, and practice of the ’Malaysian Healthy Plate’ concept among adults in Malaysia.

On the other hand, 20.4% or 4.4 million adults ever heard of this concept. Among those with awareness, 80.8% or 3.6 million correctly interpreted or understood the concept, and 19.2% or 0.8 million of them wrongly interpreted or misunderstood the concept. Among those who ever heard and understood the concept, only 70.5% (2.5 million) have been practising it daily or occasionally as shown in Fig 1.

## Discussion

Prevalence of never heard of the ’Malaysian Healthy Plate’ after three years of introduction was 79.6% or equivalent to the fourth-fifth of the adult population in Malaysia. The never heard of this healthy eating concept was significantly different by sociodemographic characteristics which are higher in a rural area, among men, elderly, Chinese and other ethnicities, divorcers or widowers, those with low education level, non-government staff, and those with lower household income group. Our finding is almost similar to the assessment on MyPlate in the United States where the fourth-firth of the American population was unknow of the visual icon after two years of introduction of MyPlate [13]. The same study also reported the unknowing of MyPlate was different by sex which is higher in men, by age group which is higher in older adults, by race which is higher in Mexican Americans, non-Hispanic blacks, and Asian Americans, and by household income which is higher in low household income group [13].

An updated Survey of the Nutritional Status of Filipino Children and Other Population Groups in 2015 reported that unknowing of Filipinos towards ‘Pinggang Pinoy’ which is a healthy food plate for Filipino was 89.4% after one year of introduction to the public [14]. They suggested that dissemination of the messages or campaign has not reached the population, particularly those residing in remote areas of the Philippines. Another population-based study among Canadian adults in 2015 toward Canada’s Food Guide (CFG) 2007 reported that the prevalence unknowing of CFG 2007 was 13.5% [15]. The same study also reported the unknowing of CFG was significantly higher among men, among marital statuses other than single or married, and among lower-income groups. However, the survey was conducted after eight years of the introduction of CFG in 2007 [15].

Our study also found that never heard of the ’Malaysian Healthy Plate’ was significantly associated with sex which is men were more likely to never hear of this concept compared to women. This finding is reasonable because a previous study in the United States reported women, generally, as the nutritional gatekeeper for the family, are more likely receptive to nutrition information compared to men [13]. Another study on gender differences in health information behaviour also reported women were more interested in nutrition information and reported considerably more active searching of health-related information, paid more attention to potential global pandemics, and were far more aware of how the items they buy everyday life influences their health than males were [16].

Other ethnicity or ethnicity other than Malay, Chinese or Indian is another significantly associated factor to never heard of Malaysian Healthy Plate. This finding was similar to a study on MyPlate also found ethnic differences where non-Hispanic-whites were more aware of MyPlate than other ethnicities in the United States [12]. A previous local study on hypertensive awareness among 11,288 adults also reported a similar finding where another ethnic group which is the majority indigenous group was less likely to be aware due to the resided in remote areas [17].

Education level was an important factor associated with never heard of ‘Malaysian Healthy Plate. Our study revealed that there were higher odds of never hearing of Malaysian Healthy Plate among adults with lower education levels. A study among United State consumers also found those with less education were less likely to have heard about MyPlate [18]. It is well known that education is a fundamental social determinant of health [19]. Education enhances health by increasing effective agency and cultivating a sense of personal control, both of which encourage and facilitate a healthy lifestyle. Education’s positive benefits are broad, cumulative, and self-replicating, and they expand through time [20].

Our study also found that non-government staff or not working individuals were about two times more likely to never hear of ‘Malaysian Healthy Plate’ compared with government staff. This could be due to the effect of continuous promotion in government platforms and several implementations in government workplaces regarding healthy eating such as ’Healthy Cafeteria’ recognition and ‘Healthy Meal Presentation during Meetings’ [21, 22]. The promotion of healthy eating was also expended to non-government and the public through many programs such as ’Clean, Safe & Healthy’ to restaurants and ‘Promotion of Healthy Nutrition at Supermarkets’ and many strategies such as mass media, social networks, posters, and banner in the public areas and also an online application named MyNutri, it probably did not reach the target yet [23–25].

The non-diabetic adults were also more likely to never hear of the Malaysian Healthy Plate concept than adults with diabetes. This may be due to diabetic patients normally receiving nutrition counselling from nutritionists or dietitians, regular visits to health clinics for medication, and more concern about their health status [26]. The previous study among diabetics and non-diabetic subjects on awareness of health promotion concludes that diabetic patients had more knowledge of health care than non-diabetic subjects [27]. People with low fruit and vegetable intake were also more likely to be unaware of the ‘Malaysian Healthy Plate. It has been expected since the ‘Malaysian Healthy Plate’ itself recommend consuming more fruits and vegetables compared to carbohydrate and protein potion [1]. However, a previous study on fruit and vegetable intake found that young adults had a basic understanding of general health, but they seemed unaware of health information and the 5-a-day campaign [28].

Apart from that, one-fifth who was ever heard of the ‘Malaysian Healthy Plate. Among that one-fifth, only 80.8% of them correctly interpreted or understood the concept. Our result is comparable to a previous study in the United Kingdom where they reported most of the British population sampled was aware of the national healthy eating campaign which is 5‐a‐day FV, but they cannot recount the messages correctly [29]. Early assessment of MyPlate among adults in the United States reported the participants knew of MyPlate but they were unsure of the guidelines MyPlate represented [1]. In our study, among those who ever heard of and understood the ‘Malaysian Healthy Plate’ only two-thirds of them have been practising the recommendations. Almost similar situation to the effectiveness of the MyPlate campaign in the United States, only 36% and 38%, of the adults with awareness of MyPlate respectively, actually use MyPlate as a guide when purchasing and preparing meals [30].

In 2020, new Malaysian Dietary Guidelines (MDG) 2020 have been published with major changes in Malaysia Food Pyramid 2020 to be match with the Malaysia Healthy Plate but this Malaysia Food Pyramid is recommendation for daily food intake as compared Malaysia Healthy Plate is recommendation for each meal’s intake. The 1^st^ group or the basement of the Malaysian Food Pyramid 2020 are vegetables and Fruits which means this food group should be consumed more compared to carbohydrates which becomes the 2^nd^ group or 2^nd^ level in the pyramid [31]. The introduction of this MDG 2020 is very much awaited, but big challenges may appear as the pandemic COVID-19 hit the world population in the same year. Unsustainable of food diversity and households’ nutrition insecurity led by the COVID-19 pandemic will ask an extraordinary approach and hard work to ensure the benefit of practising Pinggan Sihat Malaysia and Malaysian Food Pyramid 2020 obtained by the population [32].

This study also face some limitation as this is the first survey on prevalence ever heard of Malaysia Healthy Plate, we have to estimate the sample using 50% of proportion where it will give maximum number of sample need. However, the data collection team was successfully recruitedmore than minimum sample we need. Other limitation was the prevalence measure only for ‘ever heard’ which is only the surface picture of Malaysian Healthy Plate the coverage in population. The understanding and practice only among those who are ever heard. Besides, this National Health and Morbidity Survey is national level study and in future it should also measure the prevalence of understanding and practice among the Malaysian population.

## Conclusion

This is the first nationwide assessment on ‘Malaysian Healthy Plate’ after 3 years of its launching to the public. About a fourth-fifth of the adult population in Malaysia never heard of the ‘Malaysian Healthy Plate’ means the dissemination of this healthy eating info is still low. Sex, ethnicity, education level, work status, diabetes status, and fruit and vegetable intake status were factors associated with it. On the other hand, out of one-fifth who was ever heard of this concept, 80.8% of them correctly explained or understood the concept. In terms of practice, only 70.5% of those who were aware of and understood the concept have been practising it daily or occasionally. Continuous teaching and marketing through multiple channels, as well as multiagency collaboration on the promotion of the Malaysian Healthy Plate, are suggested to guarantee that this healthy eating concept can deliver successfully to the public, especially currently amid the Covid-19 epidemic.

## Abbreviations

aOR: adjusted Odd Ratio
BMI: Body mass index
DOSM: Department of Statistics Malaysia
EB: Enumeration Block
HCD: Health Care Demand
LQ: Living Quarters
MDG: Malaysian Dietary Guideline
MOH: Ministry of Health
NCD: Non-Communicable Disease
NHMS: National Health and Morbidity Survey
SDG: Sustainable Development Goals
SPSS: Statistical Package for the Social Sciences
WHO: World Health Organization
